# Synthesis, crystal structure and thermal properties of bis­(1,3-di­cyclo­hexyl­thio­urea-κ*S*)bis(iso­thiocyanato-κ*N*)cobalt(II)

**DOI:** 10.1107/S205698902101327X

**Published:** 2022-01-01

**Authors:** Christoph Krebs, Inke Jess, Christian Näther

**Affiliations:** a Institute of Inorganic Chemistry, University of Kiel, Max-Eyth.-Str. 2, 24118 Kiel, Germany

**Keywords:** crystal structure, cobalt(II)thio­cyanate, 1,3-di­cyclo­hexyl­thio­urea, thermal properties

## Abstract

Crystals of the title compound, [Co(NCS)_2_(C_13_H_24_N_2_S)_2_], were obtained by the reaction of Co(NCS)_2_ with 1,3-di­cyclo­hexyl­thio­urea in ethanol. Its crystal structure consists of discrete complexes that are located on twofold rotation axes, in which the Co^II^ cations are tetra­hedrally coordinated by two terminal N-bonded thio­cyanate anions and two 1,3-di­cyclo­hexyl­thio­urea ligands. These complexes are linked *via* inter­molecular N—H⋯S and C—H⋯S hydrogen bonding into chains, which elongate in the *b-*axis direction. These chains are closely packed in a pseudo-hexa­gonal manner. The CN stretching vibration of the thio­cyanate anions located at 2038 cm^−1^ is in agreement with only terminal bonded anionic ligands linked to metal cations in a tetra­hedral coordination. TG–DTA measurements prove the decomposition of the compound at about 227°C. DSC measurements reveal a small endothermic signal before decomposition at about 174°C, which might correspond to melting.

## Abstract 2

The crystal structure of the title compound consists of discrete tetra­hedral complexes, which are linked by inter­molecular N—H⋯S and C—H⋯S hydrogen bonding into chains.

## Chemical context

Coordination polymers based on Co(NCS)_2_ have been investigated for several years because they can show inter­esting magnetic properties due to the large magnetic anisotropy of Co^II^. This is the reason why we and others are especially inter­ested in this class of compounds. In most cases, the Co^II^ cations are octa­hedrally coordinated and linked by pairs of thio­cyanate anions into chains, even if a few compounds with single thio­cyanate bridges have been reported (Palion-Gazda *et al.*, 2015 ▸). If the Co cations are all-*trans* or *cis*–*cis*–*trans* coordinated with the thio­cyanate anions in the *trans*-position, the chains are linear and frequently show anti­ferromagnetic or ferromagnetic behavior or a slow relaxation of the magnetization indicative of single-chain magnetism (Wang *et al.*, 2005 ▸; Shurda *et al.*, 2013 ▸; Wöhlert *et al.*, 2014 ▸; Jin *et al.*, 2007 ▸; Prananto *et al.*, 2017 ▸; Mautner *et al.*, 2018 ▸; Rams *et al.*, 2020 ▸; Jochim *et al.*, 2020*a* ▸). In the case where the Co centers are *cis*–*cis*–*trans* coordinated with the thio­cyanate anions in the *cis*-position, the chains are corrugated and the magnetic exchange is suppressed (Shi *et al.*, 2007 ▸; Böhme *et al.*, 2020 ▸). In some cases Co(NCS)_2_ layers are observed, in which the Co cations are linked by single and double thio­cyanate bridges or by single anionic ligands exclusively (Suckert *et al.*, 2016 ▸; Werner *et al.*, 2015*a* ▸). These compounds usually show ferromagnetic behavior with low critical temperatures, which can be tuned by mixed crystal formation with Ni^II^ cations (Wellm *et al.*, 2018 ▸, 2020 ▸; Neumann *et al.*, 2018*a* ▸).

In the case where monocoordinating co-ligands are used and the chains are linear, these compounds have the general composition Co(NCS)_2_(*L*)_2_ (*L* = co-ligand) but for this composition a second structure exists, in which the Co cations are tetra­hedrally coordinated and in this case, no cooperative magnetic exchange inter­actions can be observed. The reason why, dependent on the nature of the co-ligand, chains or complexes are formed is not clear. First of all, one can assume that the cobalt cations would prefer a tetra­hedral coordination with bulky co-ligands because of steric crowding. On the other hand, we observed that strong N-donor co-ligands such as, for example, 4-(di­methyl­amino)­pyridine would lead to the formation of tetra­hedral complexes (Neumann *et al.*, 2018*b* ▸), whereas weaker donors such as 4-(4-chloro­benz­yl)pyridine (Werner *et al.*, 2015*b* ▸) or 4-(3-phenyl­prop­yl)pyridine (Werner *et al.*, 2014 ▸; Ceglarska *et al.*, 2021 ▸) lead to the formation of chains. In the case of inter­mediate donor ligands like 4-meth­oxy­pyridine, both isomers can be obtained, chains and discrete complexes (Mautner *et al.*, 2018 ▸; Rams *et al.*, 2020 ▸). 
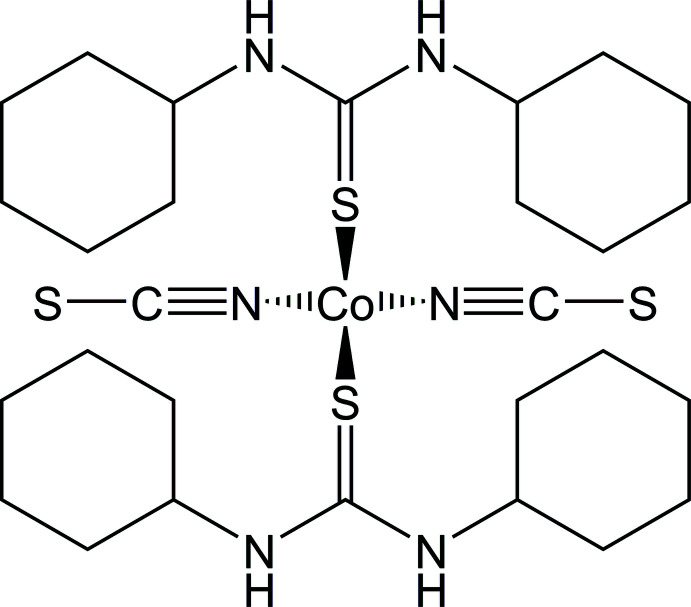


**Figure 1:**
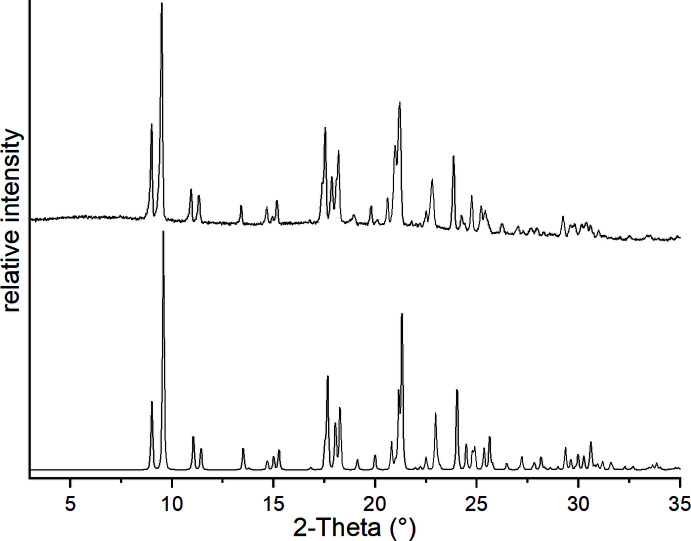
Experimental (top) and calculated powder pattern (bottom) of the title compound measured with Cu *K*α radiation.

In the course of our systematic work, we became inter­ested in S-donor co-ligands and with thio­urea we obtained a compound with the desired chain structure showing anti­ferromagnetic ordering but no slow relaxation of the magnetization (Jochim *et al.*, 2020*a* ▸). In further work, we obtained two compounds with 1,3-di­methyl­thio­urea (and 1,1,3,3-tetra­methyl­thio­urea) but in this case, tetra­hedral discrete complexes were obtained (Jochim *et al.*, 2020*b* ▸,*c* ▸). To investigate the influence of the co-ligand in more detail we used 1,3-di­cyclo­hexyl­thio­urea as the co-ligand and we obtained crystals of the title compound, which were characterized by single crystal X-ray diffraction, which proves the formation of a discrete complex even with this ligand. Investigations using X-ray powder diffraction show that the title compound was obtained as a pure phase (Fig. 1 ▸). The CN stretching vibration is observed at 2038 cm^−1^, which is typical for thio­cyanates that are only terminal bonded to metal cations in a tetra­hedral coordination (Fig. S1). Measurements using simultaneously differential thermoanalysis (DTA) and thermogravimetry reveal the decomposition of the title compound starting at about 227°C, which is accompanied with an endothermic event in the DTA curve (Fig. S2). The experimental mass loss of 37.7% is in a reasonable agreement with that calculated for the removal of one 1,3-di­cyclo­hexyl­thio­urea ligand of 36.6%. The mass loss in the second step is higher than expected for the removal of the second 1,3-di­cyclo­hexyl­thio­urea ligand, but in this temperature region the thio­cyanate anions also decompose. Additional measurements using differential scanning calorimetry show a small endothermic event before the compound decomposes (Fig. S3). To check if this event corresponds to some transition, the residue formed after the endothermic signal (see point ‘*x*′ in Fig. S3) was isolated and investigated by XRPD measurements, which shows that the powder pattern is identical to that of the pristine material but of lower crystallinity (Fig. S4).

## Structural commentary

**Figure 2:**
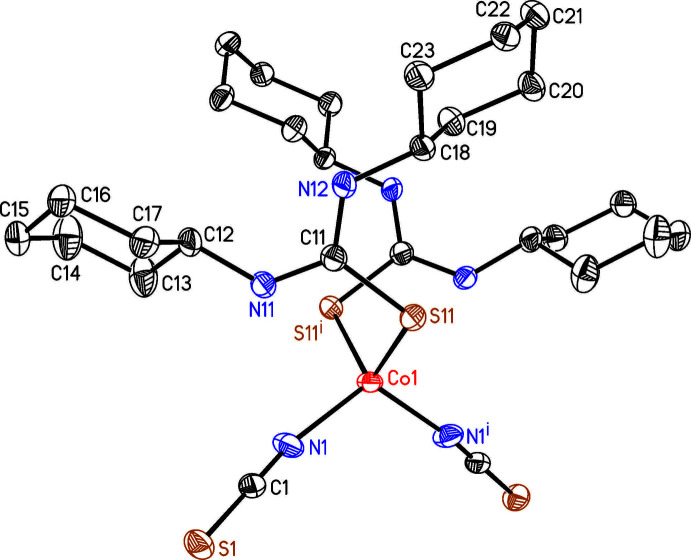
Crystal structure of the title compound with labeling and displacement ellipsoids drawn at the 50% probability level. [Symmetry code: (i) −*x* + 1, *y*, −*z* + 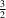 .]

**Figure 3:**
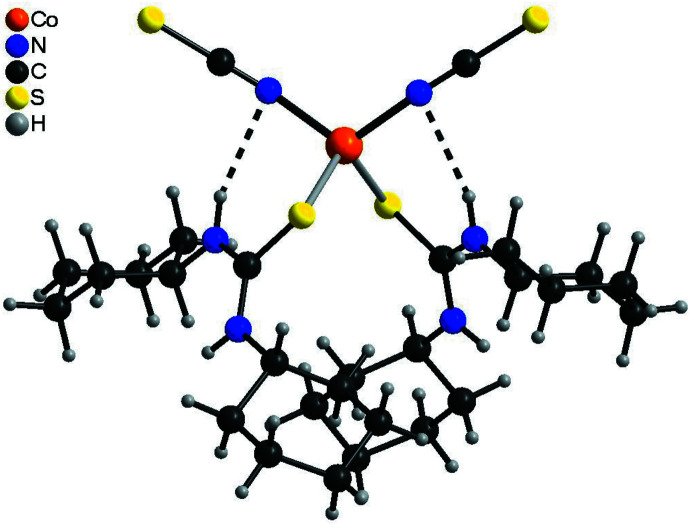
View of the discrete complex with intra­molecular N—H⋯N hydrogen bonding shown as dashed lines.

The asymmetric unit of the title compound consists of one Co^II^ cation that is located on a twofold rotation axis, one thio­cyanate anion and one 1,3-di­cyclo­hexyl­thio­urea ligand that occupies general positions. The Co^II^ cations are fourfold coordinated by two terminal N-bonded thio­cyanate anions and two sulfur atoms of 1,3-di­cyclo­hexyl­thio­urea ligands each (Fig. 2 ▸). The Co—N and Co—S distances are comparable to that observed in other Co(NCS)_2_ compounds with thio­urea derivatives (Table 1 ▸, Jochim *et al.*, 2020*a* ▸,*b* ▸). The bond angles deviate from the ideal values, revealing that the tetra­hedra are slightly distorted (see supporting information). Both hexane rings of the 1,3-di­methyl­thio­urea ligand are in a chair conformation (Figs. 2 ▸ and 3 ▸). There are two symmetry-equivalent intra­molecular N—H⋯N hydrogen bonds between the amino H atom of the 1,3-di­cyclo­hexyl­thio­urea ligand and the N atoms of the thio­cyanate anions (Table 2 ▸ and Fig. 3 ▸). The N—H⋯N angle is close to linearity, indicating that this is a relatively strong inter­action (Table 2 ▸).

## Supra­molecular features

**Figure 4:**
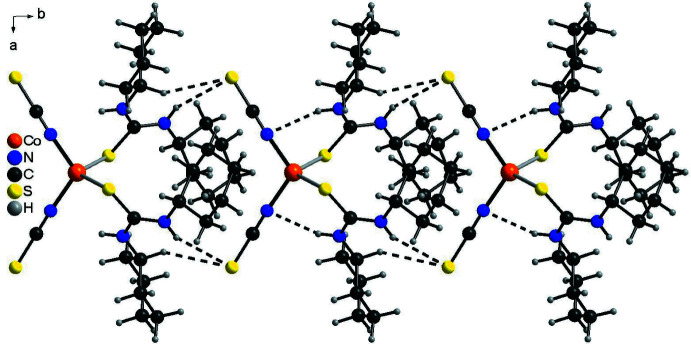
Crystal structure of the title compound with a view of a chain formed by inter­molecular N—H⋯S and C—H⋯S hydrogen bonding (dashed lines).

**Figure 5:**
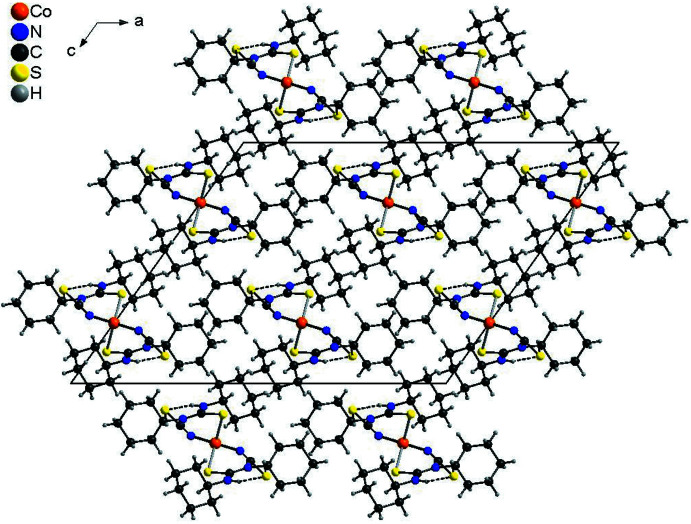
Crystal structure of the title compound with a view in the direction of the crystallographic *b*-axis, showing the arrangement of the chains. Inter­molecular N—H⋯S and C—H⋯S hydrogen bonding is shown as dashed lines.

In the crystal structure of the title compound the discrete complexes are linked into chains by two inter­molecular N—H⋯S hydrogen bonds related by the twofold rotation axis between the N—H H atoms and the thio­cyanate S atom of a neighboring complex (Fig. 4 ▸, Table 2 ▸). The discrete complexes are additionally linked by two symmetry-equivalent C—H⋯S hydrogen bonds, which might correspond to a weak inter­action (Fig. 4 ▸, Table 2 ▸). These chains elongate along the *b-*axis direction and each chain is surrounded by six neighboring chains in a pseudo-hexa­gonal manner (Fig. 5 ▸).

## Database survey

There are only ten crystal structures with this ligand reported in the Cambridge Structural Database (CSD version 5.42, last update November 2020; Groom *et al.*, 2016 ▸). The most important for us is bis­(1,3-di­cyclo­hexyl­thio­urea)bis­(iso­thio­cyanato)­zinc(II), which is isotypic to the title compound (refcode: TINBIC; Jia *et al.*, 2007 ▸). These authors also reported the structure of hexa­kis­(1,3-di­cyclo­hexyl­thio­urea)lead(II)bis­(iso­thio­cyanate) ethanol solvate, which consists of discrete complexes, in which the Pb^II^ cations are octa­hedrally coordinated by six 1,3-di­cyclo­hexyl­thio­urea ligands (refcode: TINBUO; Jia *et al.*, 2007 ▸). In that paper, the crystal structure of bis­(1,3-di­cyclo­hexyl­thio­urea)di­chloro­cobalt(II) is also reported (refcode: TINBEY). The crystal structures of chloro­bis­(1,3-di­cyclo­hexyl­thio­urea)copper(I), of bromo­bis­(1,3-di­cyclo­hexyl­thio­urea)copper(I) (refcodes: WODVER and WODVIV; Jia *et al.*, 2008 ▸) and of chloro-tris­(1,3-di­cyclo­hexyl­thio­urea)tellurium(II) chloride (refcode: OCAWUK; Husebye *et al.*, 2001 ▸) also consist of discrete complexes. The crystal structure of 1,3-di­cyclo­hexyl­thio­urea was reported by Ramnathan *et al.* (1996 ▸) (refcode: ZIVGUG).

There are also several crystal structures with Co(NCS)_2_ reported, in which the Co^II^ cations are tetra­hedrally coordinated by two terminal N-bonded thio­cyanate anions and two N-donor co-ligands, for example two polymorphic modifications of bis­(4-di­methyl­amino­pyridine)­bis­(iso­thio­cyanato)­cobalt(II) (refcode: GIQPEE; Neumann *et al.*, 2018*a* ▸; Krebs *et al.*, 2021 ▸), bis­(4-vinyl­pyridine)­di(iso­thio­cyanato)­cobalt(II) (refcode: BOZJUW; Foxman & Mazurek, 1982 ▸), bis­(2-chloro­pyridine)­bis­(iso­thio­cyanato)­cobalt(II), bis­(2-bromo­pyridine)­bis­(iso­thio­cyanato)­cobalt(II), bis­(2-methyl­pyridine)bis­(iso­thio­cyanato)­cobalt(II) (refcodes: DEYDUI, DEYFIY and DEYGAR; Wöhlert *et al.*, 2013 ▸) and bis­(4-meth­oxy­pyridine)­bis­(iso­thio­cyanato)­cobalt(II) (refcode: KIJQAY; Mautner *et al.*, 2018 ▸).

Two structures have already been reported with thio­urea derivatives and Co(NCS)_2_, *viz*. bis­(1,3-di­methyl­thio­urea)bis­(iso­thio­cyanato)­cobalt(II) (refcode: QUSZAI; Jochim *et al.*, 2020*b* ▸) and bis­(1,1,3,3-tetra­methyl­thio­urea)bis­(iso­thio­cyanato)­cobalt(II) (refcode: WUQTIO; Jochim *et al.*, 2020*c* ▸).

## Synthesis and crystallization

**Synthesis**

Co(NCS)_2_ was purchased from Merck. 1,3-Di­cyclo­hexyl­thio­urea was purchased from Alfa Aesar. All chemicals were used without further purification. Blue-colored single crystals suitable for single-crystal X-ray analysis were obtained after storage of 0.25 mmol Co(NCS)_2_ (43.8 mg) and 0.50 mmol 1,3-di­cyclo­hexyl­thio­urea (120.2 mg) in 2.0 ml ethanol at 333 K over night.

**Experimental details**

The data collection for single crystal structure analysis was performed using an XtaLAB Synergy, Dualflex, HyPix diffractometer from Rigaku with Cu-Kα radiation.

The IR spectrum was measured using an ATI Mattson Genesis Series FTIR Spectrometer, control software: *WINFIRST*, from ATI Mattson.

The PXRD measurement was performed with Cu *K*α_1_ radiation (λ = 1.540598 Å) using a Stoe Transmission Powder Diffraction System (STADI P) equipped with a MYTHEN 1K detector and a Johansson-type Ge(111) monochromator.

Thermogravimetry and differential thermoanalysis (TG–DTA) measurements were performed in a dynamic nitro­gen atmosphere in Al_2_O_3_ crucibles using a STA-PT 1000 thermobalance from Linseis. The instrument was calibrated using standard reference materials.

The DSC experiments were performed using a DSC 1 star system with STARe Excellence software from Mettler-Toledo AG under dynamic nitro­gen flow in Al pans.

## Refinement

Crystal data, data collection and structure refinement details are summarized in Table 3 ▸. All non-hydrogen atoms were refined anisotropically. The C-bound H atoms were positioned with idealized geometry and were refined isotropically with *U*_iso_(H) = 1.2 *U*_eq_(C) using a riding model.

## Supplementary Material

Crystal structure: contains datablock(s) I. DOI: 10.1107/S205698902101327X/zq2269sup1.cif

Structure factors: contains datablock(s) I. DOI: 10.1107/S205698902101327X/zq2269Isup2.hkl

Figure S1. IR spectrum of the title compound. Given is the value of the CN stretching vibration. DOI: 10.1107/S205698902101327X/zq2269sup3.png

Figure S2. DTG (top) TG (mid) and DTA curve of the title compound measured with 4C/min. Given is the mass loss in % and the peak temperature. DOI: 10.1107/S205698902101327X/zq2269sup4.png

Figure S3. DSC curve of the title compound measured with 4C/min. x denotes the point where the residue was isolated. DOI: 10.1107/S205698902101327X/zq2269sup5.png

Figure S4. Experimental XRPD pattern measured with Cu Kalpha radiation of the residue formed after the first weak endothermic signal in a DSC measurement (top) and XRPD pattern of the title compound calculated from single crystal data (bottom). DOI: 10.1107/S205698902101327X/zq2269sup6.png

CCDC reference: 2128608

Additional supporting information:  crystallographic information; 3D view; checkCIF report

## Figures and Tables

**Table 1:** Selected geometric parameters (Å, °)

Co1—N1	1.9516 (16)	Co1—S11	2.3130 (5)
			
N1—Co1—N1^i^	113.00 (10)	N1—Co1—S11	106.00 (5)
N1—Co1—S11^i^	109.67 (5)		

Symmetry code: (i) -x+1, y, -z+{\script{3\over 2}}.

**Table 2:** Hydrogen-bond geometry (Å, °)

*D*—H⋯*A*	*D*—H	H⋯*A*	*D*⋯*A*	*D*—H⋯*A*
N11—H11⋯N1	0.88	2.33	3.169 (2)	160
C12—H12⋯S1^ii^	1.00	2.93	3.774 (2)	143
N12—H12*A*⋯S1^ii^	0.88	2.84	3.6770 (16)	159
C19—H19*B*⋯S11	0.99	3.00	3.529 (2)	114

Symmetry code: (ii) x, y-1, z.

**Table 3:** Experimental details

Crystal data	
Chemical formula	[Co(NCS)_2_(C_13_H_24_N_2_S)_2_]
*M*_r_	655.89
Crystal system, space group	Monoclinic, *C*2/*c*
Temperature (K)	100
*a*, *b*, *c* (Å)	24.0667 (4), 8.8282 (1), 18.8910 (3)
β (°)	125.619 (2)
*V* (Å^3^)	3262.76 (11)
*Z*	4
Radiation type	Cu *K*α
μ (mm^−1^)	6.73
Crystal size (mm)	0.15 × 0.08 × 0.03

Data collection	
Diffractometer	XtaLAB Synergy, Dualflex, HyPix
Absorption correction	Multi-scan (*CrysAlis PRO*; Rigaku OD, 2021 ▸)
*T*_min_, *T*_max_	0.704, 1.000
No. of measured, independent and observed [*I* > 2σ(*I*)] reflections	20399, 3503, 3462
*R*_int_	0.025
(sin θ/λ)_max_ (Å^−1^)	0.639

Refinement	
*R*[*F* ^2^ > 2σ(*F* ^2^)], *wR*(*F* ^2^), *S*	0.035, 0.096, 1.05
No. of reflections	3503
No. of parameters	177
H-atom treatment	H-atom parameters constrained
Δρ_max_, Δρ_min_ (e Å^−3^)	0.65, −0.36

Computer programs: *CrysAlis PRO* (Rigaku OD, 2021 ▸), *SHELXT2014/5* (Sheldrick, 2015*a* ▸), *SHELXL2016/6* (Sheldrick, 2015*b* ▸), *DIAMOND* (Brandenburg & Putz, 1999 ▸) and *publCIF* (Westrip, 2010 ▸).

## supplementary crystallographic information

### Crystal data

**Table:**

[Co(NCS)_2_(C_13_H_24_N_2_S)_2_]	*F*(000) = 1396
*M**_r_* = 655.89	*D*_x_ = 1.335 Mg m^−^^3^
Monoclinic, *C*2/*c*	Cu *K*α radiation, λ = 1.54184 Å
*a* = 24.0667 (4) Å	Cell parameters from 13904 reflections
*b* = 8.8282 (1) Å	θ = 2.9–78.5°
*c* = 18.8910 (3) Å	µ = 6.73 mm^−^^1^
β = 125.619 (2)°	*T* = 100 K
*V* = 3262.76 (11) Å^3^	Block, intense blue
*Z* = 4	0.15 × 0.08 × 0.03 mm

### Data collection

**Table:**

XtaLAB Synergy, Dualflex, HyPix diffractometer	3503 independent reflections
Radiation source: micro-focus sealed X-ray tube, PhotonJet (Cu) X-ray Source	3462 reflections with *I* > 2σ(*I*)
Mirror monochromator	*R*_int_ = 0.025
Detector resolution: 10.0000 pixels mm^-1^	θ_max_ = 80.0°, θ_min_ = 4.5°
ω scans	*h* = −30→30
Absorption correction: multi-scan (CrysAlisPro; Rigaku OD, 2021)	*k* = −11→10
*T*_min_ = 0.704, *T*_max_ = 1.000	*l* = −20→24
20399 measured reflections	

### Refinement

**Table:**

Refinement on *F*^2^	Primary atom site location: dual
Least-squares matrix: full	Hydrogen site location: inferred from neighbouring sites
*R*[*F*^2^ > 2σ(*F*^2^)] = 0.035	H-atom parameters constrained
*wR*(*F*^2^) = 0.096	*w* = 1/[σ^2^(*F*_o_^2^) + (0.054*P*)^2^ + 5.0479*P*] where *P* = (*F*_o_^2^ + 2*F*_c_^2^)/3
*S* = 1.05	(Δ/σ)_max_ = 0.001
3503 reflections	Δρ_max_ = 0.65 e Å^−^^3^
177 parameters	Δρ_min_ = −0.36 e Å^−^^3^
0 restraints	

### Special details

**Table:**

Geometry. All esds (except the esd in the dihedral angle between two l.s. planes) are estimated using the full covariance matrix. The cell esds are taken into account individually in the estimation of esds in distances, angles and torsion angles; correlations between esds in cell parameters are only used when they are defined by crystal symmetry. An approximate (isotropic) treatment of cell esds is used for estimating esds involving l.s. planes.

### Fractional atomic coordinates and isotropic or equivalent isotropic displacement parameters (Å^2^)

**Table:**

	*x*	*y*	*z*	*U*_iso_*/*U*_eq_	
Co1	0.500000	0.91248 (4)	0.750000	0.02073 (12)	
N1	0.57874 (8)	1.03449 (18)	0.78063 (11)	0.0283 (3)	
C1	0.62890 (10)	1.1041 (2)	0.82549 (13)	0.0241 (4)	
S1	0.69885 (2)	1.19936 (6)	0.88857 (3)	0.02946 (13)	
S11	0.53579 (2)	0.76716 (5)	0.87146 (3)	0.02478 (12)	
C11	0.59547 (9)	0.6489 (2)	0.87525 (12)	0.0221 (3)	
N11	0.63300 (8)	0.70206 (18)	0.84996 (11)	0.0257 (3)	
H11	0.628830	0.799458	0.837934	0.031*	
C12	0.68063 (9)	0.6182 (2)	0.83960 (13)	0.0250 (4)	
H12	0.664260	0.511209	0.823464	0.030*	
C13	0.67862 (11)	0.6900 (3)	0.76500 (14)	0.0377 (5)	
H13A	0.691074	0.798367	0.777936	0.045*	
H13B	0.631610	0.683568	0.710954	0.045*	
C14	0.72780 (12)	0.6107 (3)	0.75124 (15)	0.0433 (6)	
H14A	0.712524	0.504987	0.732404	0.052*	
H14B	0.727319	0.662885	0.704507	0.052*	
C15	0.80009 (11)	0.6105 (3)	0.83419 (15)	0.0342 (5)	
H15A	0.817213	0.715832	0.849578	0.041*	
H15B	0.830355	0.553156	0.824289	0.041*	
C16	0.80239 (10)	0.5391 (2)	0.90921 (14)	0.0297 (4)	
H16A	0.849393	0.546680	0.963165	0.036*	
H16B	0.790582	0.430304	0.896754	0.036*	
C17	0.75272 (10)	0.6167 (2)	0.92332 (13)	0.0276 (4)	
H17A	0.752748	0.562352	0.969193	0.033*	
H17B	0.767965	0.721974	0.943308	0.033*	
N12	0.60327 (7)	0.50735 (18)	0.90351 (10)	0.0224 (3)	
H12A	0.635837	0.452301	0.908415	0.027*	
C18	0.56066 (9)	0.4371 (2)	0.92713 (12)	0.0212 (3)	
H18	0.550070	0.515812	0.955748	0.025*	
C19	0.49330 (9)	0.3789 (2)	0.84687 (12)	0.0244 (4)	
H19A	0.502584	0.301113	0.817278	0.029*	
H19B	0.468264	0.463403	0.805576	0.029*	
C20	0.44955 (10)	0.3106 (2)	0.87343 (13)	0.0262 (4)	
H20A	0.437460	0.390552	0.898999	0.031*	
H20B	0.406622	0.270325	0.821191	0.031*	
C21	0.48772 (10)	0.1834 (2)	0.93957 (13)	0.0281 (4)	
H21A	0.495221	0.098353	0.911865	0.034*	
H21B	0.459693	0.145614	0.958672	0.034*	
C22	0.55656 (10)	0.2392 (2)	1.01863 (12)	0.0256 (4)	
H22A	0.581609	0.153099	1.058555	0.031*	
H22B	0.548684	0.315490	1.050304	0.031*	
C23	0.60031 (10)	0.3096 (2)	0.99230 (13)	0.0264 (4)	
H23A	0.643242	0.349894	1.044513	0.032*	
H23B	0.612306	0.231184	0.965832	0.032*	

### Atomic displacement parameters (Å^2^)

**Table:**

	*U*^11^	*U*^22^	*U*^33^	*U*^12^	*U*^13^	*U*^23^
Co1	0.0151 (2)	0.0140 (2)	0.0277 (2)	0.000	0.00945 (17)	0.000
N1	0.0226 (8)	0.0191 (8)	0.0361 (9)	−0.0028 (6)	0.0130 (7)	0.0022 (7)
C1	0.0240 (9)	0.0176 (8)	0.0317 (9)	0.0029 (7)	0.0167 (8)	0.0038 (7)
S1	0.0212 (2)	0.0271 (2)	0.0354 (2)	−0.00593 (17)	0.0138 (2)	−0.00315 (18)
S11	0.0221 (2)	0.0209 (2)	0.0337 (2)	0.00480 (16)	0.01759 (19)	0.00516 (17)
C11	0.0167 (8)	0.0214 (9)	0.0251 (8)	0.0010 (7)	0.0105 (7)	0.0026 (7)
N11	0.0209 (7)	0.0206 (8)	0.0381 (9)	0.0044 (6)	0.0186 (7)	0.0086 (6)
C12	0.0182 (8)	0.0251 (9)	0.0329 (9)	0.0022 (7)	0.0156 (8)	0.0053 (8)
C13	0.0241 (10)	0.0537 (14)	0.0336 (10)	0.0071 (9)	0.0157 (9)	0.0142 (10)
C14	0.0342 (12)	0.0667 (17)	0.0342 (11)	0.0050 (11)	0.0229 (10)	0.0070 (11)
C15	0.0251 (10)	0.0360 (11)	0.0479 (12)	0.0000 (8)	0.0250 (10)	0.0022 (9)
C16	0.0199 (9)	0.0290 (10)	0.0376 (10)	0.0010 (8)	0.0154 (8)	−0.0003 (8)
C17	0.0234 (9)	0.0285 (9)	0.0292 (9)	0.0023 (7)	0.0144 (8)	0.0011 (8)
N12	0.0177 (7)	0.0215 (7)	0.0298 (7)	0.0023 (6)	0.0148 (6)	0.0042 (6)
C18	0.0186 (8)	0.0197 (8)	0.0261 (9)	0.0001 (7)	0.0134 (7)	0.0027 (7)
C19	0.0221 (9)	0.0261 (9)	0.0242 (8)	−0.0031 (7)	0.0129 (7)	−0.0002 (7)
C20	0.0227 (9)	0.0275 (10)	0.0290 (9)	−0.0066 (7)	0.0155 (8)	−0.0030 (7)
C21	0.0324 (10)	0.0239 (9)	0.0344 (10)	−0.0052 (8)	0.0232 (9)	−0.0014 (8)
C22	0.0280 (9)	0.0238 (9)	0.0288 (9)	0.0040 (7)	0.0188 (8)	0.0066 (7)
C23	0.0220 (9)	0.0255 (9)	0.0310 (9)	0.0044 (7)	0.0151 (8)	0.0086 (7)

### Geometric parameters (Å, º)

**Table:**

Co1—N1	1.9516 (16)	C16—H16B	0.9900
Co1—N1^i^	1.9517 (16)	C16—C17	1.530 (3)
Co1—S11	2.3130 (5)	C17—H17A	0.9900
Co1—S11^i^	2.3131 (5)	C17—H17B	0.9900
N1—C1	1.167 (3)	N12—H12A	0.8800
C1—S1	1.620 (2)	N12—C18	1.472 (2)
S11—C11	1.7431 (18)	C18—H18	1.0000
C11—N11	1.330 (2)	C18—C19	1.526 (2)
C11—N12	1.328 (2)	C18—C23	1.525 (2)
N11—H11	0.8800	C19—H19A	0.9900
N11—C12	1.470 (2)	C19—H19B	0.9900
C12—H12	1.0000	C19—C20	1.529 (2)
C12—C13	1.520 (3)	C20—H20A	0.9900
C12—C17	1.522 (3)	C20—H20B	0.9900
C13—H13A	0.9900	C20—C21	1.526 (3)
C13—H13B	0.9900	C21—H21A	0.9900
C13—C14	1.522 (3)	C21—H21B	0.9900
C14—H14A	0.9900	C21—C22	1.528 (3)
C14—H14B	0.9900	C22—H22A	0.9900
C14—C15	1.519 (3)	C22—H22B	0.9900
C15—H15A	0.9900	C22—C23	1.534 (3)
C15—H15B	0.9900	C23—H23A	0.9900
C15—C16	1.522 (3)	C23—H23B	0.9900
C16—H16A	0.9900		
			
N1—Co1—N1^i^	113.00 (10)	C12—C17—C16	110.88 (16)
N1—Co1—S11^i^	109.67 (5)	C12—C17—H17A	109.5
N1—Co1—S11	106.00 (5)	C12—C17—H17B	109.5
N1^i^—Co1—S11^i^	106.00 (5)	C16—C17—H17A	109.5
N1^i^—Co1—S11	109.67 (5)	C16—C17—H17B	109.5
S11—Co1—S11^i^	112.63 (3)	H17A—C17—H17B	108.1
C1—N1—Co1	157.11 (17)	C11—N12—H12A	118.1
N1—C1—S1	179.39 (19)	C11—N12—C18	123.87 (15)
C11—S11—Co1	101.24 (6)	C18—N12—H12A	118.1
N11—C11—S11	119.32 (14)	N12—C18—H18	108.3
N12—C11—S11	120.02 (13)	N12—C18—C19	111.41 (15)
N12—C11—N11	120.67 (16)	N12—C18—C23	109.67 (14)
C11—N11—H11	116.0	C19—C18—H18	108.3
C11—N11—C12	127.98 (16)	C23—C18—H18	108.3
C12—N11—H11	116.0	C23—C18—C19	110.89 (16)
N11—C12—H12	108.5	C18—C19—H19A	109.7
N11—C12—C13	107.87 (16)	C18—C19—H19B	109.7
N11—C12—C17	111.80 (16)	C18—C19—C20	110.05 (15)
C13—C12—H12	108.5	H19A—C19—H19B	108.2
C13—C12—C17	111.64 (16)	C20—C19—H19A	109.7
C17—C12—H12	108.5	C20—C19—H19B	109.7
C12—C13—H13A	109.4	C19—C20—H20A	109.4
C12—C13—H13B	109.4	C19—C20—H20B	109.4
C12—C13—C14	111.01 (18)	H20A—C20—H20B	108.0
H13A—C13—H13B	108.0	C21—C20—C19	110.96 (16)
C14—C13—H13A	109.4	C21—C20—H20A	109.4
C14—C13—H13B	109.4	C21—C20—H20B	109.4
C13—C14—H14A	109.4	C20—C21—H21A	109.5
C13—C14—H14B	109.4	C20—C21—H21B	109.5
H14A—C14—H14B	108.0	C20—C21—C22	110.86 (16)
C15—C14—C13	111.2 (2)	H21A—C21—H21B	108.1
C15—C14—H14A	109.4	C22—C21—H21A	109.5
C15—C14—H14B	109.4	C22—C21—H21B	109.5
C14—C15—H15A	109.4	C21—C22—H22A	109.2
C14—C15—H15B	109.4	C21—C22—H22B	109.2
C14—C15—C16	111.08 (17)	C21—C22—C23	111.83 (16)
H15A—C15—H15B	108.0	H22A—C22—H22B	107.9
C16—C15—H15A	109.4	C23—C22—H22A	109.2
C16—C15—H15B	109.4	C23—C22—H22B	109.2
C15—C16—H16A	109.3	C18—C23—C22	109.63 (15)
C15—C16—H16B	109.3	C18—C23—H23A	109.7
C15—C16—C17	111.61 (17)	C18—C23—H23B	109.7
H16A—C16—H16B	108.0	C22—C23—H23A	109.7
C17—C16—H16A	109.3	C22—C23—H23B	109.7
C17—C16—H16B	109.3	H23A—C23—H23B	108.2

Symmetry code: (i) −*x*+1, *y*, −*z*+3/2.

### Hydrogen-bond geometry (Å, º)

**Table:**

*D*—H···*A*	*D*—H	H···*A*	*D*···*A*	*D*—H···*A*
N11—H11···N1	0.88	2.33	3.169 (2)	160
C12—H12···S1^ii^	1.00	2.93	3.774 (2)	143
N12—H12*A*···S1^ii^	0.88	2.84	3.6770 (16)	159
C19—H19*B*···S11	0.99	3.00	3.529 (2)	114

Symmetry code: (ii) *x*, *y*−1, *z*.
