# Supplementary figures and images for: Synthesis, crystal structure and thermal properties of bis­(1,3-di­cyclo­hexyl­thio­urea-κS)bis(iso­thiocyanato-κN)cobalt(II)
Source: Acta Crystallogr E Crystallogr Commun. 2022 Jan 1;78(Pt 1):71–5. doi: 10.1107/S205698902101327X (PMC8739206; doi:10.1107/S205698902101327X)

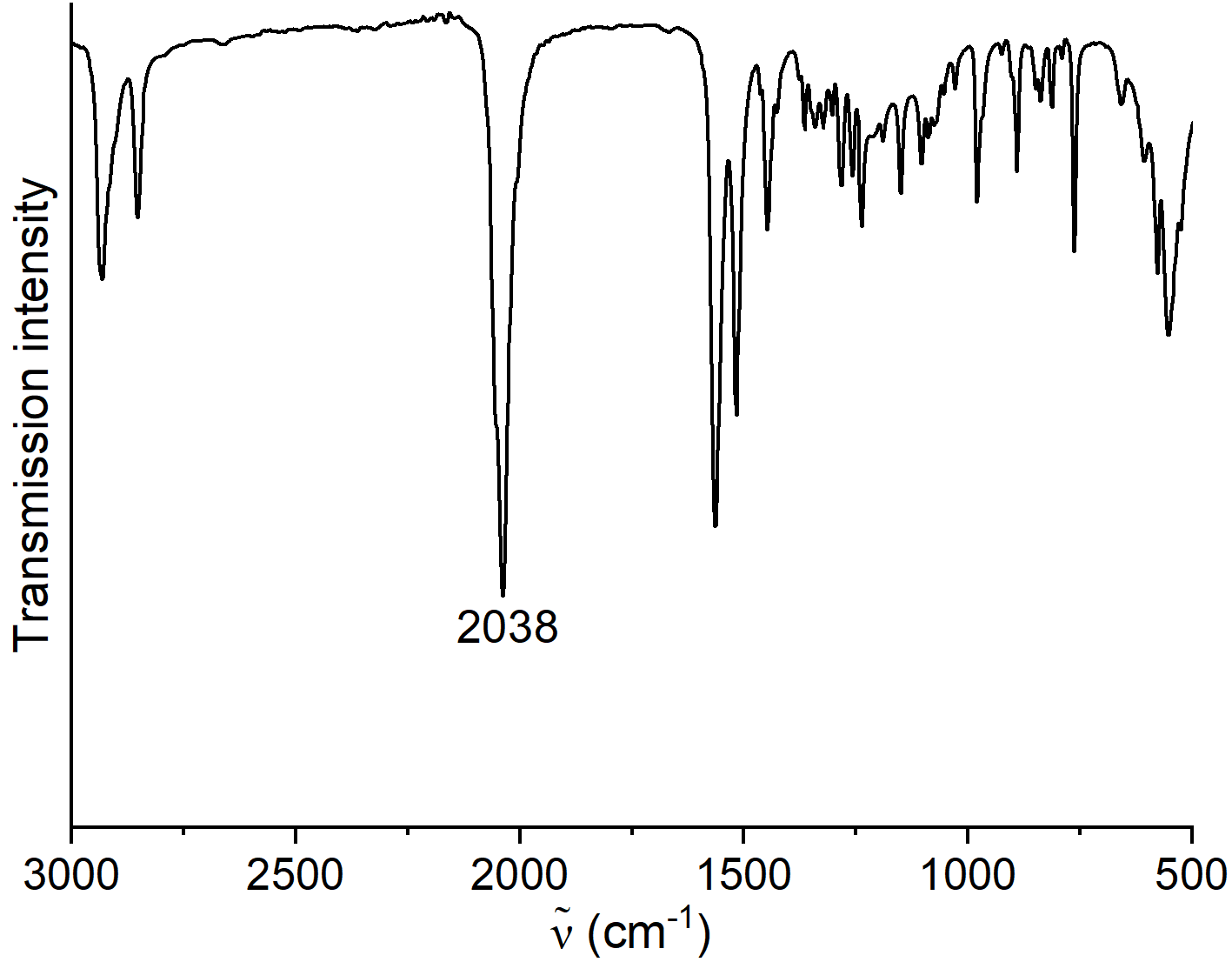

Supplement: Supplementary file 3 [file e-78-00071-sup3.png]

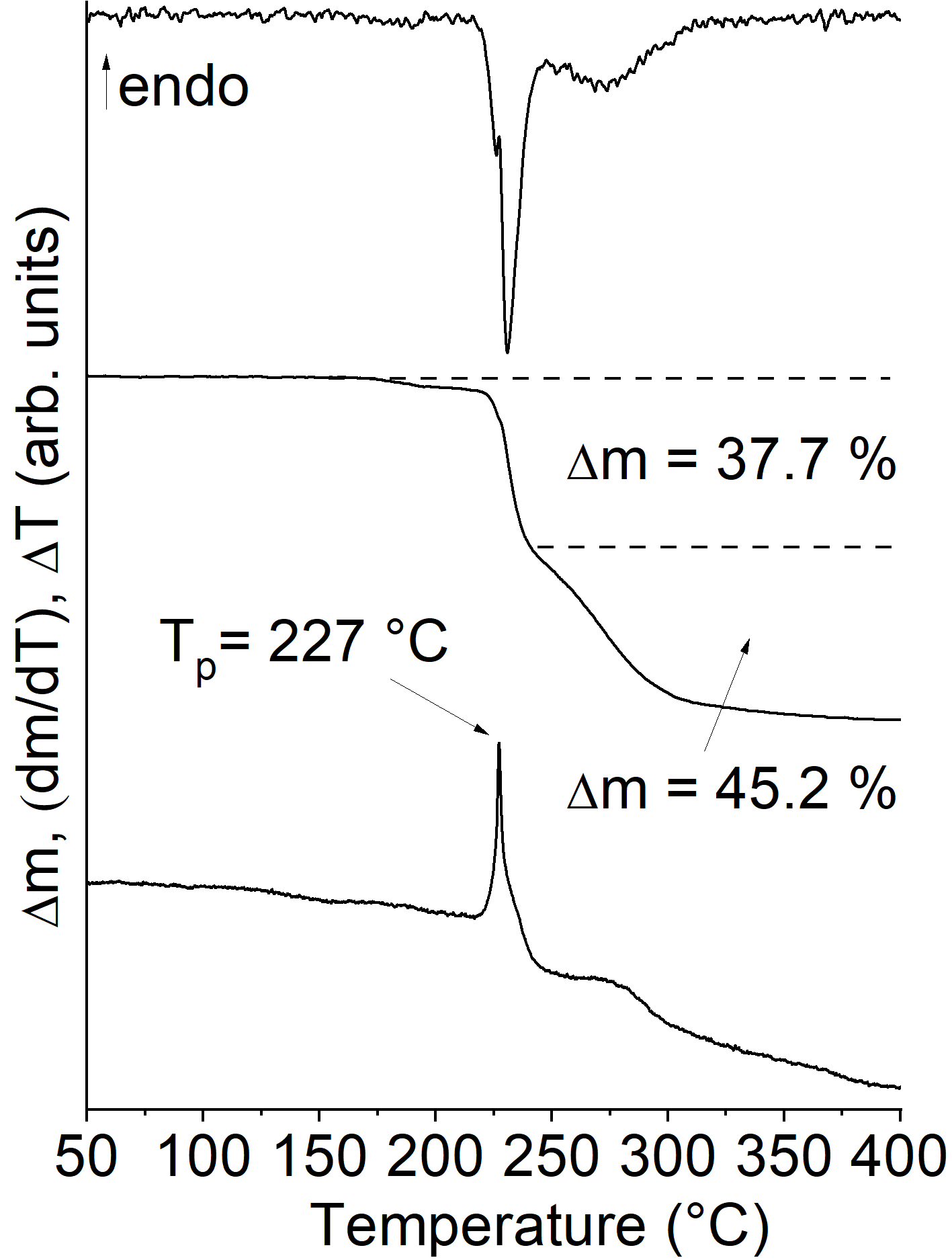

Supplement: Supplementary file 4 [file e-78-00071-sup4.png]

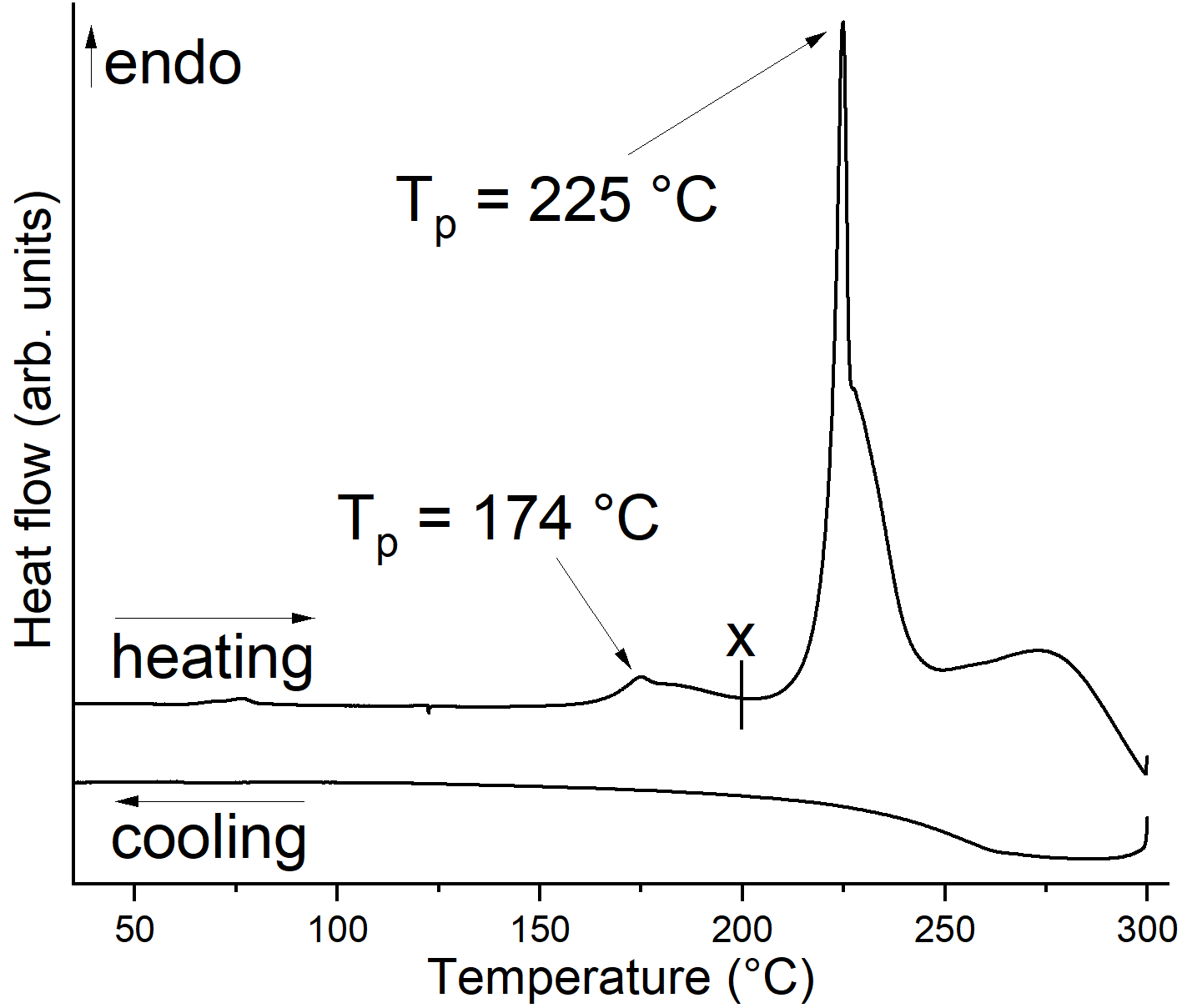

Supplement: Supplementary file 5 [file e-78-00071-sup5.png]

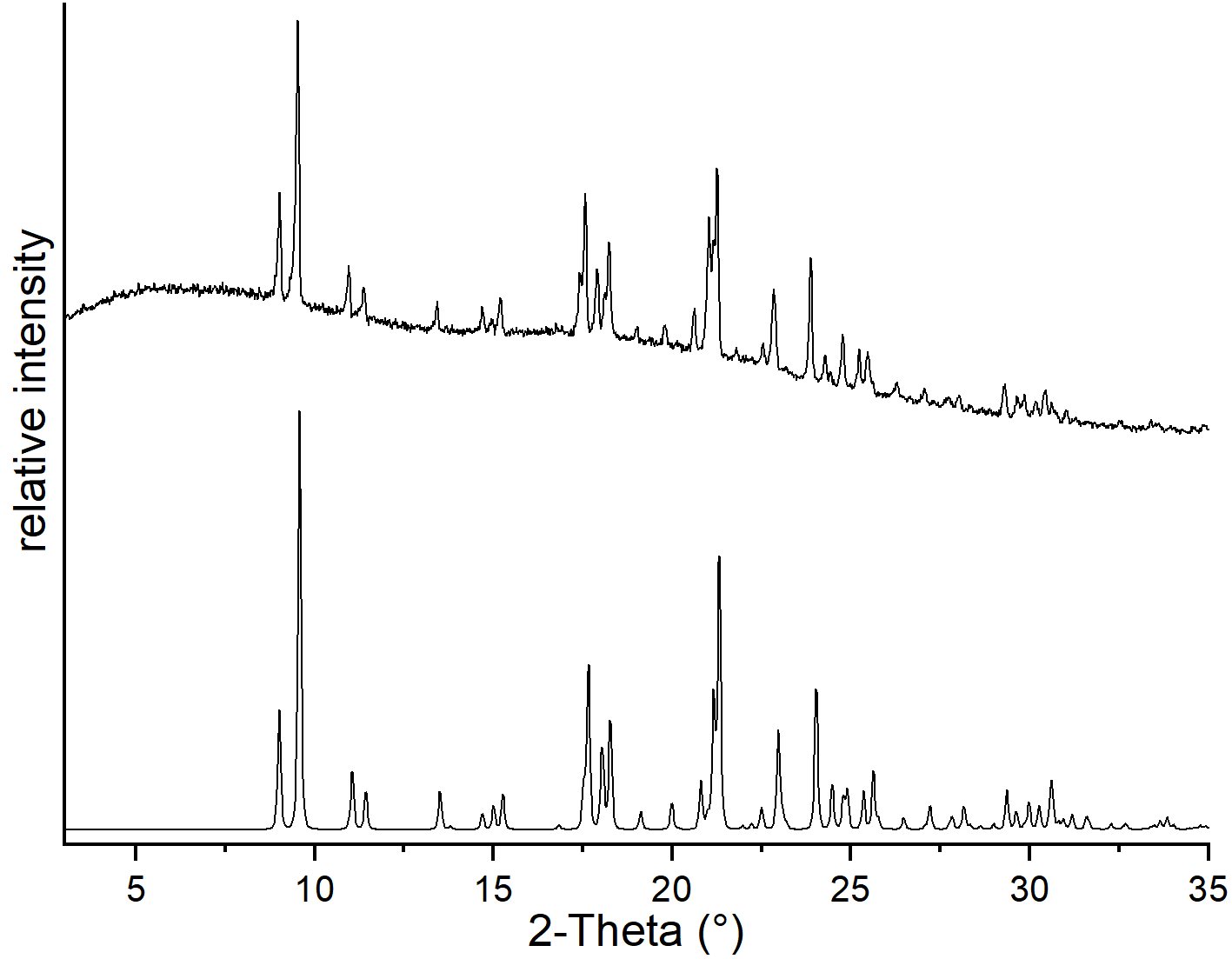

Supplement: Supplementary file 6 [file e-78-00071-sup6.png]
